# Quantifying the anatomical variability of the proximal femur

**DOI:** 10.1007/s11548-025-03560-5

**Published:** 2025-12-19

**Authors:** Angelika Ramesh, Johann Henckel, Alister Hart, Anna Di Laura

**Affiliations:** 1 Department of Mechanical Engineering, University College London, London, UK; 2 Royal National Orthopaedic Hospital NHS Trust, Stanmore, UK; 3 Institute of Orthopaedics and Musculoskeletal Science, University College London, London, UK; 4 Cleveland Clinic London, London, UK; 5 Department of Mechanical Engineering, University College London, London, UK

**Keywords:** Proximal femur, Femoral version, Statistical shape modelling, Total hip arthroplasty, Intramedullary variability, Preoperative planning

## Abstract

**Purpose:**

Achieving a prosthetic femoral version (PFV) within the target range of 10–20° is crucial for optimal biomechanics in total hip arthroplasty (THA). Predicting the PFV preoperatively is challenging due to the limited understanding of the relationship between native femoral version (NFV) and the morphology of the intramedullary canal. This study aims to quantify the 3D morphological variability and identify the most variable anatomical features of the proximal femur pre- and post-operatively.

**Methods:**

Pre- and post-operative CT scans from 62 patients (31 males, 31 females) who underwent THA and received a single stem design (straight, triple-tapered) were analysed. Four femoral models were generated per patient: 1. Native proximal femur, 2. Native femur after neck osteotomy, 3. Internal femoral canal after neck osteotomy, and 4. Reconstructed femur. Statistical Shape Models (SSMs) were developed separately by sex, and principal component analysis (PCA) was used to identify dominant modes of anatomical variation.

**Results:**

The first three principal components (PCs) accounted for over 60% of shape variability across all models. PFV showed weak correlation with NFV as variability existed between the SSM of the internal femoral canal and SSM of the native proximal femur. Sex-specific differences in the measured NFV and PFV were found, with females exhibiting a greater range and a more anteverted femur/femoral stem. The female canal model showed intramedullary version variability; however, this variability was not present in the first three PCs in the corresponding male model.

**Conclusions:**

This study demonstrates that PFV cannot be reliably predicted from NFV alone. These findings underscore the need for advanced, 3D preoperative planning tools to better predict stem version and accommodate patient-specific anatomy. Additionally, the increased variability observed in females may warrant sex-specific consideration in implant design choice and surgical technique.

## Introduction

In cementless total hip arthroplasty (THA), it is often inaccurately assumed that the native femoral version (NFV) directly correlates with prosthetic femoral version (PFV) [1, 2]. In practice, these stems achieve press-fit fixation dictated by the anatomy of the femoral canal [3]. Due to the large variability in the shape of the intramedullary cavity, the range of PFV achieved varies considerably across patients, reportedly from − 17° retroversion to 60° anteversion [4]. If the stem orientation could be accurately determined before surgery, the decision between cemented and uncemented fixation could be made on a case-by-case basis.

To address the broad spectrum of proximal femoral morphology, varying designs of cementless femoral stems have evolved over the years [5]. This includes stems with modular neck options [6], monoblock stems with a built-in anteversion/retroversion [7], and also short stems [8]. While these options intend to give the orthopaedic surgeon more independent rotational control and flexibility over the PFV, they may not always be a suitable option to restore the optimal biomechanics of the hip.

Uncertainty remains in the prediction of the PFV [9]. There is limited focus on the shape of the internal femoral canal during the planning of these prostheses, and currently no tool exists which can accurately predict the PFV [10, 11].

The aim of the present study is to quantify the 3D morphological variability and identify the most variable anatomical features of the proximal femur pre- and post-operatively. The primary objective was to develop a sex-specific statistical shape model (SSM) of the internal and external geometry of both the native and reconstructed proximal femur. The secondary objective was to use the model to correlate the top three principal components (PCs) to anatomical features relevant to the femur, by quantifying shape changes across the modes of variation.

## Materials and methods

### Study population

This is a retrospective study involving 62 (31 male, 31 female) pre- and post-operative Computed Tomography (CT) scans of patients who underwent THA. All patients received a single design of femoral stem; the Quadra®–H stem (Medacta International, Switzerland), a straight, triple-tapered, cementless femoral stem type. The indication for surgery was osteoarthritis (OA) for all patients. The mean age at the time of surgery for female patients was 66 ± 11 years and 60 ± 13 years for the male patients.

### Surgical procedure

All surgical procedures were performed by a single consultant orthopaedic surgeon using the posterior approach. A commercially available, patient-specific femoral neck osteotomy guide (MyHip, Medacta International, Switzerland) was used intraoperatively to perform the neck cut according to the preoperative plan (in terms of both the osteotomy angle and level).

A measure of NFV was provided preoperatively by the manufacturer, and in some patients, this demonstrated either excessive or insufficient anteversion of the femoral neck. In all cases, the surgeon aimed to achieve a PFV of 20° ± 5°. Despite this target, the ability to adjust stem rotation intraoperatively was limited, and no further adjustments could be made.

### Three-dimensional dataset preparation

**Fig. 1:**
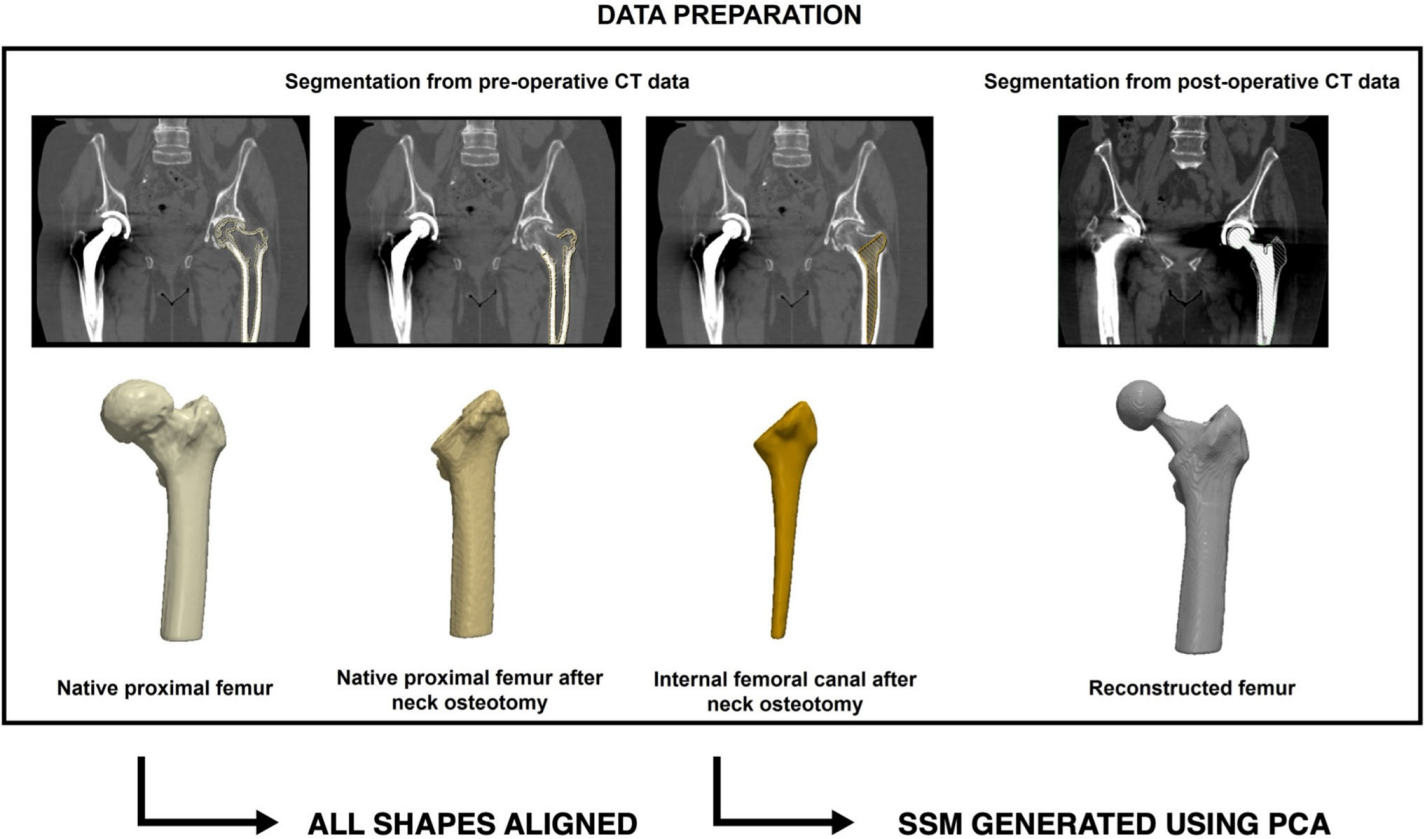
Study workflow for building the statistical shape model (SSM) of the four different regions of interest (ROI)—the native proximal femur, the native femur after neck osteotomy, the internal femoral canal after neck osteotomy, and the reconstructed femur (from the post-operative CT scan). After segmenting the ROIs from each patient’s CT data, the shapes were aligned using a common coordinate system. Principal component analysis (PCA) was then performed to generate a shape model which shows the surface deviations relative to a reference geometry

To develop a statistical shape model (SSM) of the proximal femur, 3D reconstructions of the femoral anatomy were generated using Simpleware ScanIP Medical (version 2024.12, Synopsys, Mountain View, CA, USA)– a medical image processing software solution. Four different regions of interest were segmented from each patient’s CT scan (Fig. 1): the native proximal femur, the native femur after neck osteotomy, the internal femoral canal after neck osteotomy (all segmented from the preoperative scan), and the reconstructed femur (from the corresponding post-operative scan).

For the segmentation of the native proximal femur (model 1), a combination of thresholding and manual segmentation was used (threshold between 230 Hounsfield Units (HU) to maximum on HU scale [~1000 HU]). To obtain a 3D reconstruction of the native femur after neck osteotomy (model 2), the stereolithography (STL) file of the planned osteotomy cut was imported and co-registered to proximal femur on the correct side, before simulating the neck cut on the model. The femoral neck osteotomy was simulated using the preoperatively planned cut plane for all cases, ensuring that the model closely reflected the surgically intended osteotomy, which serves as the basis for predicting the PFV. In all 62 cases, the osteotomy was executed using a patient-specific femoral osteotomy guide. The accuracy of these guides has been validated in a previous 3D-CT study which included the patients in this series [12] and demonstrated median discrepancies of 0.2° (IQR −1° to 3°) in neck cut angle and 0.3 mm (IQR −1 mm to 2 mm) in cut level. These findings support the use of the planned cutting plane as a reliable approximation of the achieved osteotomy, giving confidence that the simulated cut closely reflects the actual cut in this cohort. Additionally, this method was selected because the planned neck cut model (STL) eliminates metal artefacts that would otherwise be present on post-operative CT scans.

A Boolean subtraction between this model (model 2) and a completely filled proximal femur resulted in a 3D representation of the intramedullary cavity (model 3). Finally, to build model 4, metal and bone were separately segmented, and only the femoral stem was kept from the metal segmentation, as this was the component of interest in this model. The femoral stem and proximal femur were then grouped to obtain a single object surface.

Each segmented shape was standardised for length (~15 cm) and surface roughness to ensure consistent input surfaces into the SSM. To further standardise the dataset, all right-sided femurs were mirrored along the y-axis, simulating a left-sided femur.

### Alignment of the surfaces

For each CT scan, the scanner coordinate system was replaced with a surgically relevant coordinate system, defined by the line extending from the intertrochanteric crest to the midpoint of the posterior condyles, and the posterior condylar axis [13, 14]. This ensures that all surfaces can be aligned in a common frame of reference.

Once defining this common reference frame for each pre- and post-operative CT scan, all comparable shapes were then rigidly aligned to bring them into a common space, using translation, without any rotation or scaling. Translation was used to accommodate for different patient heights.

### Statistical shape modelling

After the initial gross alignment of the shapes, each femoral surface was discretised in order to represent the surface using a set of dense points. An average of these discrete points allowed for a mean reference geometry to be computed for each model. This is used as a baseline for comparing shape variations. Using the dense points, point correspondence could be established between each surface and this reference surface, as each coordinate/point represents the same feature across all shapes. Within Simpleware Scan IP, principal component analysis (PCA) was performed to identify the main shape variations of each training surface from the reference shape. The results from this analysis were summarised and outputted in the form of PC modes—each mode describes a particular (or combination of) shape variable(s) within the population (directions of maximum variance, in other words, the eigenvectors). These modes are ranked in descending order based on the percentage of variance captured by each (degree of variance in the direction of the eigenvector—the eigenvalue). The PC modes could be visualised in the outputted shape model by changing the weights of each mode from − 3 standard deviations (SD) to +3SD. This illustrated the main anatomic differences between the reference geometry and the training surfaces in the population.

### Interpretation of the results

To associate each PC to a known shape variable/parameter, the deviation of the shape from the reference shape along each of the first three principal modes/components was measured. Feature-based measurements were taken to objectively correlate each mode to an anatomical feature. This was achieved by generating shapes at ± 3SD for each of the first three PCs and then measuring the appropriate morphometric features relevant to the proximal femur on each of these shapes. Based on the expertise of the orthopaedic surgeons and engineers involved in this study and upon visual inspection of the modes, the features of interest were size (overall and head size), femoral version, varus/valgus orientation, femoral neck shaft angle, vertical femoral offset (VFO), and horizontal femoral offset (HFO). For models 2 and 3, the shapes did not include the femoral head. Therefore, in these models, a measure of the axial rotation was used, whereby the orientation of the line connecting the most medial and lateral aspect on the face of the osteotomy cut plane relative to the posterior condylar axis was measured.

After measuring these parameters for the reference shape and the ± 3SD shapes generated for the first three PCs in each model, the measurements were used to label each PC. This was done by assigning the most prominently changing feature to each mode.

To better understand intra-individual differences between the NFV and PFV, the analysis of this was also conducted based on each patient’s CT scan (pre- and post-operatively).

## Results

### SSM of the native proximal femur

**Fig. 2:**
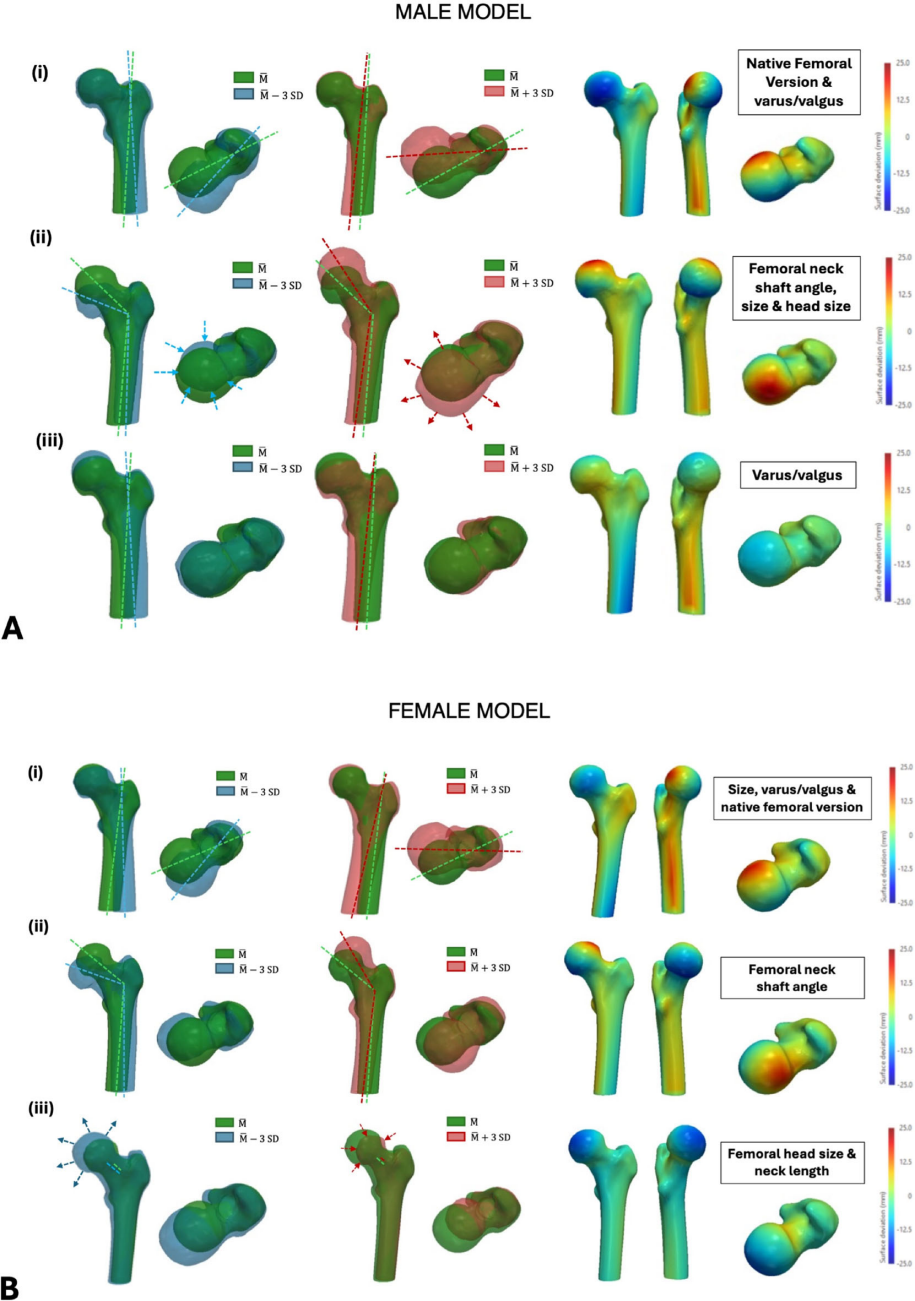
Results from the first SSM of the native proximal femur for both the male (**A**) and female (**B**) patient cohort, showing the first 3 PCs. The respective deviations from the reference geometry ($\overline{\mathrm{M} }$) are shown: (i) First PC; (ii) Second PC; (iii) Third PC. The colourmaps are represented on the reference shape and red/blue regions represent maximum deviations from the reference shape (in opposing directions)

For the male patient cohort, the first three principal components (PCs) in the SSM of the native proximal femur accounted for 78% of the total variance in the dataset (PC1 = 37%, PC2 = 28%, PC3 = 13%). From visual and feature-based analysis (Fig. 2), the first PC showed changes in the NFV and varus/valgus orientation of the proximal femur. The second PC revealed changes in a combination of features, including size (both overall size and size of the femoral head) and femoral neck shaft angle. Variations in the varus/valgus angulation of the proximal femur also showed up as the main variable in the third PC. For the female patient cohort, the first three PCs in the SSM of the native proximal femur accounted for 80% of the total variance in the dataset (PC1 = 42%, PC2 = 27%, PC3 = 11%). The first PC showed combined changes in size, varus/valgus orientation and the NFV. The second PC generated variations in the femoral neck shaft angle, and the third PC showed more localized deformations at the femoral head and neck, revealing changes in head size and femoral neck length.

### SSM of the native proximal femur after neck osteotomy

**Fig. 3:**
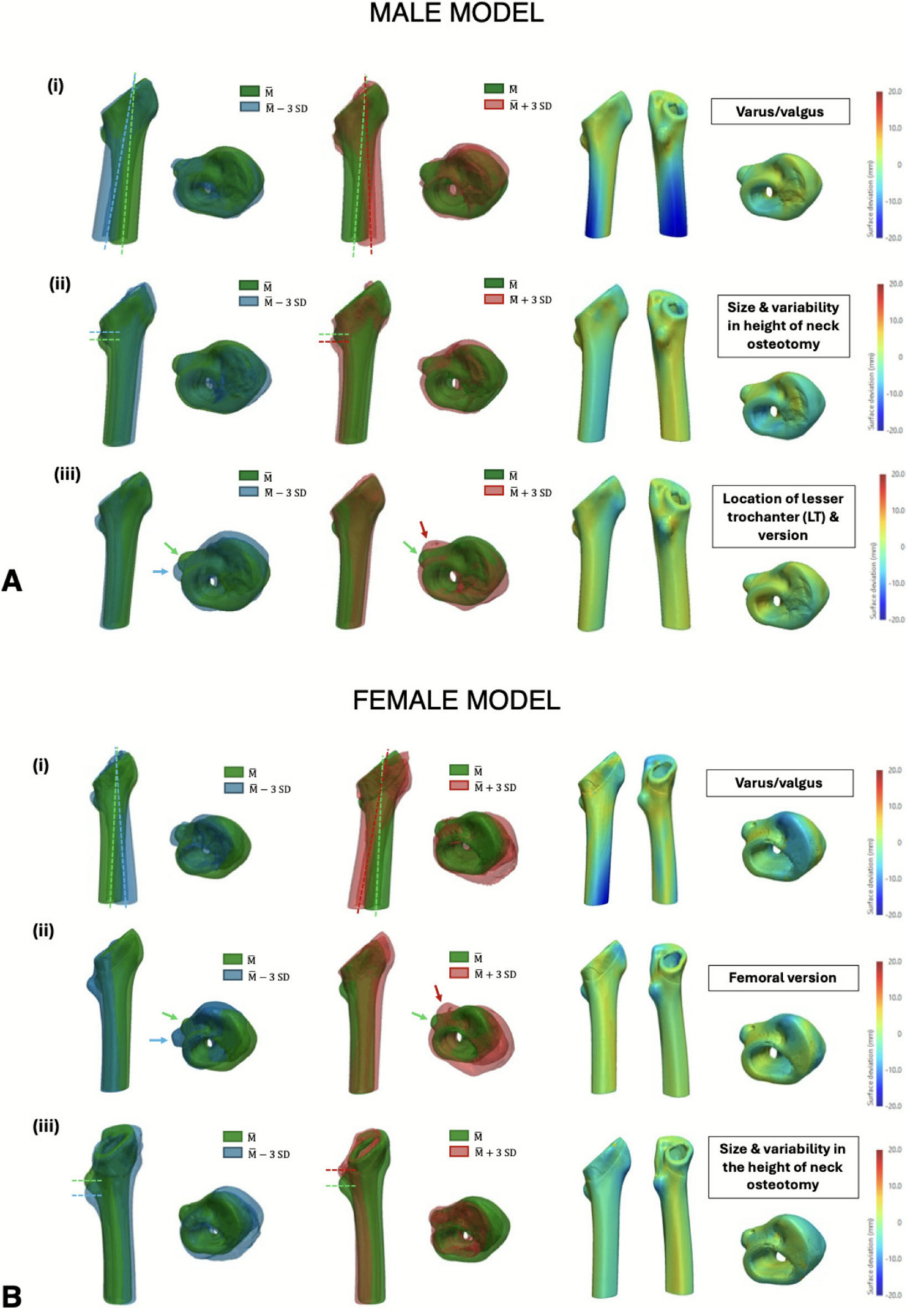
Results from the second SSM of the native femur after neck osteotomy for both the male (**A**) and female (**B**) patient cohort, showing the first 3 PCs. The respective deviations from the reference geometry ($\overline{\mathrm{M} }$) are shown: (i) First PC; (ii) Second PC; (iii) Third PC. The colourmaps are represented on the reference shape and red/blue regions represent maximum deviations from the reference shape (in opposing directions)

For the male patient cohort, the first three PCs in the SSM of the native proximal femur after neck osteotomy accounted for 66% of the total variance in the dataset (PC1 = 33%, PC2 = 22%, PC3 = 11%). The first PC demonstrated changes in the varus/valgus orientation, the second PC revealed changes in a combination of size and height of the neck osteotomy. The third PC captured variability in version and the location of the lesser trochanter (LT) (Fig. 3). For the female patient cohort, the first three PCs in the SSM of the native proximal femur after neck osteotomy accounted for 71% of the total variance in the dataset (PC1 = 34%, PC2 = 25%, PC3 = 12%). This model captured the same prominent features as in the male model, with the hierarchy of the shape variables being different. The first PC showed varus/valgus changes, the second captured variability in the femoral version, and the third PC generated size changes along with differences in the height of the neck osteotomy.

### SSM of the internal femoral canal after neck osteotomy

**Fig. 4:**
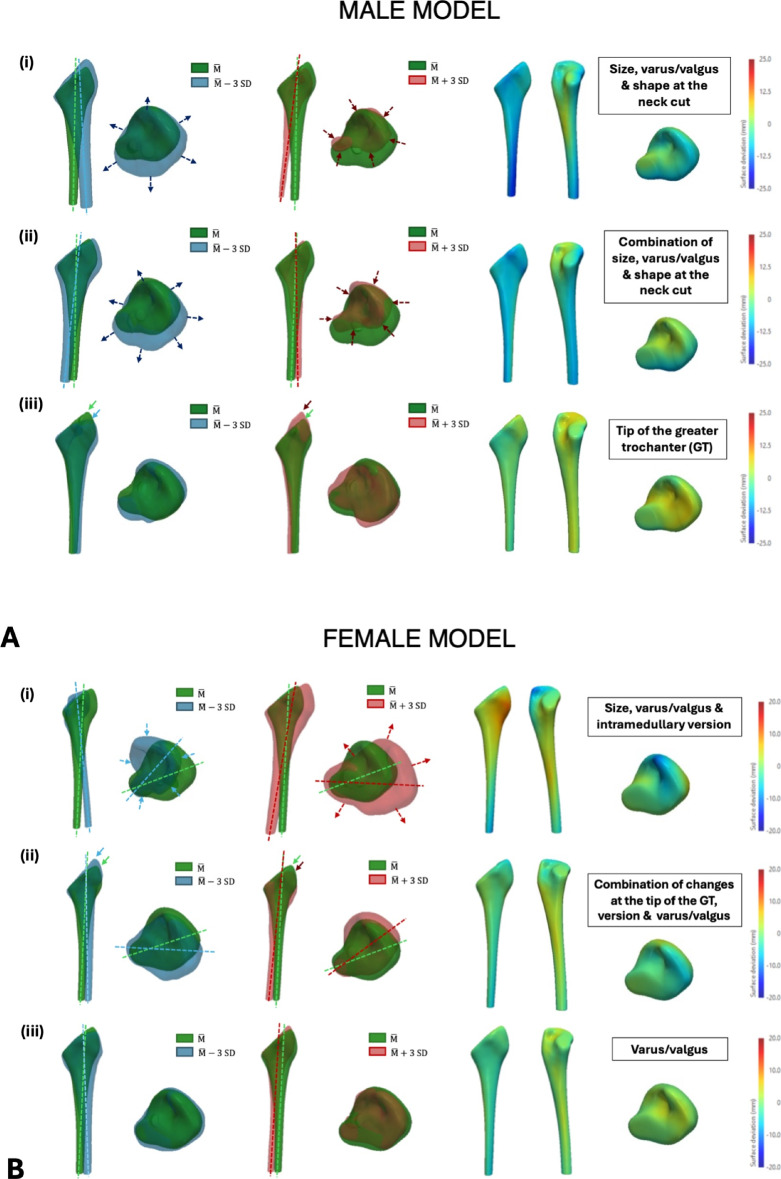
Results from the third SSM of the internal femoral canal after neck osteotomy for both the male (**A**) and female (**B**) patient cohort, showing the first 3 PCs. The respective deviations from the reference geometry ($\overline{\mathrm{M} }$) are shown: (i) First PC; (ii) Second PC; (iii) Third PC. The colourmaps are represented on the reference shape and red/blue regions represent maximum deviations from the reference shape (in opposing directions)

For the male patient cohort, the first three PCs in the SSM of the internal femoral canal after neck osteotomy accounted for 66% of the total variance in the dataset (PC1 = 33%, PC2 = 19%, PC3 = 14%). The first PC is associated with a change in the size of the femoral canal, varus/valgus orientation and variability in the shape of the face at the osteotomy cut (Fig. 4). PC2 shows a complex combination of changes in the same three variables (size, varus/valgus and neck cut shape); however, no single feature was most prominently changing in this PC (these variables are more prominently changing in PC1). PC3 mainly revealed localized changes at the tip of the greater trochanter (GT). For the female patient cohort, the first three PCs in the SSM of the internal femoral canal after neck osteotomy accounted for 74% of the total variance in the dataset (PC1 = 40%, PC2 = 22%, PC3 = 12%). The first PC generated changes in the size of the femoral canal, the varus/valgus orientation and the proximal intramedullary version. Once again, the second PC exhibited a complex combination of changes in different features, including the location of the tip of the GT, proximal intramedullary version (fixed at the calcar region) and varus/valgus. PC3 also showed some subtle varus/valgus changes, although this feature was more prominently changing in PC1.

### SSM of the reconstructed femur

**Fig. 5:**
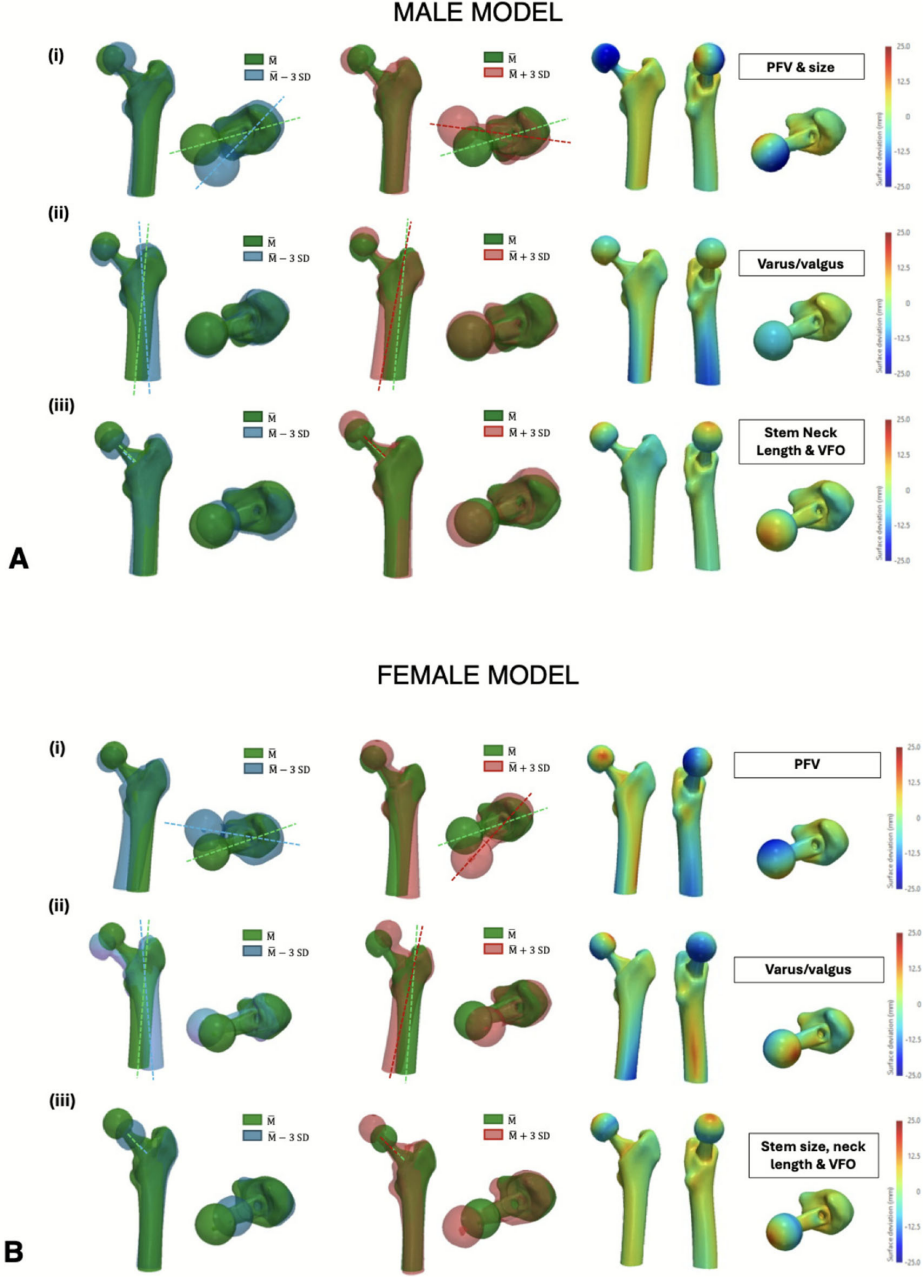
Results from the fourth statistical shape model (SSM) showing the first 3 principal components from the PCA of the reconstructed femur for both the male (**A**) and female (**B**) patient cohort. The respective deviations from the reference geometry ($\overline{\mathrm{M} }$) are shown: (i) First PC; (ii) Second PC; (iii) Third PC. The colourmaps are represented on the reference shape and red/blue regions represent maximum deviations from the reference shape (in opposing directions)

For the male patient cohort, the first three principal components (PCs) in the SSM of the reconstructed femur accounted for 71% of the total variance in the dataset (PC1 = 39%, PC2 = 21%, PC3 = 11%). The first PC is associated with PFV changes and size (size of the entire construct and also changes in stem size). The second mode of variation showed varus/valgus orientational changes, while the third PC generated changes in the femoral stem neck length and in the VFO (Fig. 5). For the female patient cohort, the first three PCs in the SSM of the reconstructed femur accounted for 79% of the total variance in the dataset (PC1 = 37%, PC2 = 26%, PC3 = 16%). The main deformation explained by the first PC was a change in the PFV. The second PC demonstrated varus/valgus changes, and the third PC showed variability in a combination of features including femoral stem size, neck length and VFO.

The morphometric features measured in each of the SSMs are summarised in Table 1, showing similarities and differences between the measurements taken across each model and between the two sexes.

**Table 1:** Summary of the morphometric measurements taken for the femur from each of the SSMs, allowing for inter-model and inter-sex comparisons

MODEL Feature	MALE MODEL			FEMALE MODEL		
**Native Proximal Femur**	− **3 SD**	**Mean**	**+3 SD**	− **3 SD**	**Mean**	**+3 SD**
NFV (°)	28	7	− 13	38	15	− 9
Varus/valgus (°)	0	7	12	-2	7	16
Femoral neck shaft angle (°)	105	125	137	110	132	139
Size (cm^3^)	239	264	324	250	181	126
Head radius (cm)	2	3	3	3	2	2
VFO (cm)	6	5	5	4	5	6
**Native Femur After Neck Osteotomy**	− **3 SD**	**Mean**	**+3 SD**	− **3 SD**	**Mean**	**+3 SD**
Neck cut version (°)	29	7	-9	29	10	-11
Varus/valgus (°)	14	7	0	− 1	7	16
Size (cm^3^)	136	128	118	111	85	68
**Internal Femoral Canal After Neck Osteotomy**	− **3 SD**	**Mean**	**+3 SD**	− **3 SD**	**Mean**	**+3 SD**
Intramedullary version (°)	----	----	----	33	8	− 10
Varus/valgus (°)	1	7	13	− 1	7	15
Size (cm^3^)	109	60	28	33	41	77
**Reconstructed Femur**	− **3 SD**	**Mean**	**+3 SD**	− **3 SD**	**Mean**	**+3 SD**
PFV (°)	43	18	− 8	− 12	15	44
Varus/valgus (°)	− 1	6	14	− 2	7	16
Femoral neck shaft angle (°)	142	132	124	127	133	144
Size (cm^3^)	216	203	245	115	142	174
Stem size (cm^3^)	6	6	8	4	5	7
VFO (cm)	5	6	7	4	6	7

**‘----'** is used in boxes where the feature did not appear as prominently changing within the first three PCs of the model

### Intraindividual differences

Having measured NFV and PFV from the pre- and post-operative CT scans of each patient included in this study, respectively, intraindividual differences could be studied. The mean ± SD NFV was 19° ± 9°, and the mean ± SD PFV was 15° ± 10°. The mean absolute difference between the version of the native femoral neck and prosthetic femoral neck was 7° ± 5°. The difference between NFV and PFV was statistically significant (*p* < 0.05), which necessitates this further study of the intramedullary anatomy of the proximal femur. Preoperative knowledge of solely the external bony anatomy (NFV) is insufficient.

## Discussion

This study sought to summarise the main shape variations of the proximal femur. The rationale behind building four separate models for each patient cohort (male and female) was to illustrate the real-life variability that orthopaedic surgeons face intraoperatively, enabling direct comparison between native and prosthetic femoral version (NFV vs PFV).

The longevity of the femoral component in THA is strongly dependent upon the alignment of the implant [9, 15]. When considering uncemented femoral stems, the orthopaedic surgeon has limited rotational control over the PFV, as these implants achieve press-fit fixation and are guided into a best-fitting position determined by the internal cavity [16]. This, combined with the inability to accurately predict the final PFV, can lead to problems such as excessive anteversion or retroversion of the femoral stem [16] in cases where cementless fixation is chosen for patients with excessive and insufficient intramedullary version.

Post-operative hip joint instability has been associated with component malpositioning [17]. A stem with correct anteversion (between 10 and 20°) [18, 19] maintains the femoral head within the acetabular cup. If excessively anteverted or retroverted, there is a greater risk of anterior or posterior impingement and dislocation, respectively [3, 19]. For the patient, this has implications of pain and restricted range of motion [18, 19]. Implant longevity is also affected by improper PFV as the physiological load distribution and forces transmitted to the implant and surrounding bone may be uneven, potentially leading to edge loading and accelerated wear. Muscle function and efficiency may also be compromised.

Cross-model analysis of the SSM results demonstrates the lack of correlation between the internal and external anatomy of the femur. This more thoroughly defines the problem as the differences in the shape of the internal femoral canal and external anatomy of the native femur can now be visualised using statistical analysis. To the authors’ best knowledge, this is the first study to illustrate these modes of variation. The differences between the NFV and PFV are highlighted in the results from model 1 (native proximal femur) and model 4 (reconstructed femur), with the PFV exhibiting a greater range than the former. This further questions the use of the NFV to guide the PFV. Therefore, inter-model differences exist, reinforcing the need to consider the shape and torsion of the internal femoral canal during 3D surgical planning.

Although it is acknowledged in the literature that the internal femoral canal dictates the final position of a cementless femoral stem [16], there is little appreciation and study of the shape of the canal and how it can be used to better predict the PFV. Though there are similarities in the morphometric features that are generated by each of the four models, the patterns of variation differ and there are clear anatomical differences between the external and internal shapes. This further emphasises the need to discard the sole use of the NFV to inform PFV. If a direct correlation between the models did exist, the preoperative prediction of PFV would not pose a challenge, as the patterns of variation in NFV would directly reflect in the PFV. However, the discrepancies between PFV and NFV reported in this study and other authors such as Hirata et al. [2] suggest otherwise.

When comparing the model of the native proximal femur with the model of the internal femoral canal after neck osteotomy, there are differences in the patterns of variation as well as in the range of the NFV and intramedullary version. In the male internal femoral canal model, intramedullary version is not identified as a key variable, whereas NFV is the most prominently changing feature in the first model for the male patient cohort. These differences emphasize the dissimilarities in the internal and external geometry of the proximal femur, particularly in terms of femoral version. When considering other features, such as the varus/valgus orientation, there is minimal variation across the models.

Another goal of this study was to study sex differences in the femoral morphology. This analysis revealed that in terms of the varus/valgus alignment of the femur, the female patient cohort showed a range from − 2° varus to 16° valgus (with the mean shape being 7° valgus). On the other hand, the male patient cohort exhibited a range from 0°, in other words neutral alignment, to 12° valgus (mean shape was 7° valgus). Therefore, the average native femoral anatomy for both sexes was 7° valgus, relative to the vertical line. The anatomical axis of the femur is known to be around 5°–7° valgus relative to the mechanical axis, so our results are in line with existing evidence [20].

The range of both the NFV and PFV measured from the SSMs was greater for the female patient cohort (NFV from 9° retroverted to 38° anteverted, PFV from 12° retroverted to 44° anteverted) compared to the male patient cohort (NFV from 13° retroverted to 28 degrees anteverted, PFV from 8° retroverted to 43° anteverted). For the mean femoral anatomy, the NFV was also measured to be greater in the female patient cohort (15°) compared with the males (7°), which is supported by several studies. Lerch et al. [21] reported a mean femoral version of 15° for male patients and 22° for female patients in their study looking at the prevalence of femoral version abnormalities in symptomatic hips attributed to Femoroacetabular Impingement (FAI) or hip dysplasia. Although their patient cohorts are different, similar patterns in the NFV were found, suggesting a tendency for the femur to be more anteverted in females compared with males. Additionally, Chadayammuri et al. [22] reported a significantly (*p* < 0.001) greater mean femoral torsion in their female patient cohort compared with the males (17° versus 9°).

Another key finding from this study was that intramedullary version was not prominently changing within the first three modes for the male cohort but is evidently changing in the first PC in the female model. This is suggestive of a greater variability in the axial rotation of the intramedullary cavity in females than males; this patient cohort may require additional considerations when choosing the fixation method (cemented versus uncemented) as there is a greater risk of the femoral stem being seated in a position not dictated by the external geometry.

The sex differences identified through these SSMs could potentially better inform surgical planning, technique and prosthetic design.

To overcome the challenge of limited rotational control in THA, several strategies have been introduced to assist orthopaedic surgeons in achieving the desired PFV. Modular neck femoral stems offer intraoperative flexibility to adjust leg length, femoral offset, and stem version [6, 23, 24]. However, concerns about modular junction failure, fretting, and corrosion ultimately led to their recall [23–25]. Short stems are now increasingly favoured in cementless primary THA due to their focus on metaphyseal fixation rather than diaphyseal engagement, allowing for some degree of proximal version adjustment [26, 27]. Nonetheless, their versatility is limited [28, 29], and the ability to fine-tune PFV heavily depends on the stem’s design. Rectangular cross sections, common in these implants, tend to occupy the proximal canal fully, reducing adjustability. Alternative approaches include modified broaching techniques or opting for cemented fixation. While these methods aim to enhance the surgeon’s control over femoral component orientation, they do not directly address the unmet need for better planning and prediction of stem version.

The authors of this study acknowledge limitations, the main limitation being the limited sample size. Firstly, the 62 patients are a relatively small patient cohort for building SSMs, in order to capture broad anatomical variability. Therefore, the results should be viewed and interpreted with caution and should not be generalised to larger, more diverse populations (e.g. patients with other hip abnormalities). However, we ensured the inclusion of a balanced patient cohort between the sexes, to avoid any skewness or bias of the data. Secondly, in the models where the femoral head was not included (models 2 and 3), version had to be approximated using surrogate landmarks on the osteotomy plane. This may introduce inaccuracies, which we aimed to minimise by systematically picking the most medial and lateral point on the face of the neck post-osteotomy to define its orientation relative to the posterior condylar axis.

## Conclusion

SSM proved valuable in highlighting the importance of preoperative assessment of the femoral canal in cementless THA. This study aimed to quantify the 3D morphological variability of the proximal femur pre- and post-operatively and identify the most variable anatomical features. The results revealed sex-specific differences in the morphology of the native proximal femur, internal femoral canal and in the achieved PFV. Despite capturing similar morphometric features, external and internal femoral anatomy showed no direct correlation, indicating that NFV is not a reliable predictor of stem alignment. Instead, greater emphasis should be placed on the intramedullary anatomy. By identifying key anatomical variations, SSM may enhance PFV prediction and support more personalised stem selection.
